# Absence of Sex-Specific Circadian Variations in Strength Among Military Cadets: A Cross-Sectional Study

**DOI:** 10.3390/jcm14207254

**Published:** 2025-10-14

**Authors:** Manuel Barea-Rodríguez, Pablo Galan-Lopez, Lennert Goossens, Rafael A. Casuso, Jesús R. Huertas

**Affiliations:** 1 Faculty of Physical Activity and Sports, Universidad Loyola Andalucía, 41704 Dos Hermanas, Spain; mbarea@uloyola.es (M.B.-R.); pgalan@uloyola.es (P.G.-L.); lgoossens@uloyola.es (L.G.); 2 Department of Physiology, University of Granada, 18012 Granada, Spain; jhuertas@ugr.es

**Keywords:** circadian rhythms, chronotype, strength, military training

## Abstract

**Background**: Circadian rhythms play a fundamental role in the development and production of strength. It is thought that highly physically active populations, such as military personnel, may not be affected by these variations. **Objectives**: This cross-sectional study examined strength and jump performance in military cadets at the beginning (08:30 a.m.) and end (02:30 p.m.) of their workday. **Methods**: Twenty females and twenty-three males performed a squat jump, countermovement jump, handgrip strength test, and isometric leg extension strength test on two separate occasions. **Results**: Two-way ANOVA showed no significant effects of time (all *p* > 0.28; partial η^2^ < 0.02) or time × sex interactions (all *p* > 0.52). Morning–afternoon differences were trivial across outcomes, ranging from −2.2 to 6.8 units (e.g., CMJ: Δ = −1.3 cm [95% CI: −3.7, 1.1]; handgrip: Δ = −0.9 kg [−3.2, 1.3]). By contrast, large sex effects were consistently observed: men outperformed women in jump height (SJ: d = 1.77; CMJ: d = 1.86), leg extension strength (d = 1.59–1.78), and handgrip strength (d = 2.94–3.08), with partial η^2^ values up to 0.71. **Conclusions**: The present study provides evidence that while sex-related differences in strength and jump performance are robust and large, time-of-day has a negligible influence on these measures in military cadets.

## 1. Introduction

Circadian rhythms are internal biological cycles that follow a pattern of approximately 24 h and regulate various physiological and behavioural functions in humans. They affect sleep, wakefulness, body temperature, hormone production, and metabolism [1]. In addition, many studies have analyzed the effects on the variation in force production as a function of the time of day the measurement was taken [2]. Muscle strength tends to be higher in the afternoon and early evening (between 02:00 p.m. and 08:00 p.m.) due to higher body temperature and better neuro-muscular efficiency [3]. In the morning, strength tends to be lower due to lower body temperature and muscle stiffness. Therefore, athletes tend to perform better in strength and power tests in the afternoon, when body temperature and hormone levels, such as testosterone, are more favourable [4].

Regarding neural factors, neural activation capacity and neuromuscular efficiency also follow a circadian pattern, showing greater efficiency in the afternoon. Hormones such as cortisol and testosterone, which influence energy and recovery capacity, show circadian variations that affect sporting performance [5]. Fatigue perception is usually lower, and recovery capacity is faster in the afternoon, favouring better performance in high-intensity activities [6]. In addition, circadian rhythms may significantly impact injury occurrence through their effects on physical performance, coordination and hormone secretion. Physical performance, including muscular strength and endurance, is higher in the afternoon, suggesting that training in the afternoon may reduce the risk of fatigue-related injuries [7]. Motor coordination and reaction time also improve in the afternoon, reducing the likelihood of errors and injuries [8]. Furthermore, hormones that affect muscle growth and recovery, such as testosterone and cortisol, follow circadian patterns, with cortisol levels highest during the morning to help mobilize energy and testosterone peaking during the morning to promote protein synthesis and muscle recovery [9].

Maintaining elevated physical fitness levels constitutes a fundamental requirement for military personnel’s effectiveness [10,11,12]. Achievement of requisite physical conditioning standards necessitates the implementation of daily resistance training protocols and compound exercise regimens. Optimal physical fitness represents a critical determinant for military populations, functioning not merely as a foundation for general physiological health but as an essential component for enhanced training adaptations, injury risk mitigation, and operational performance capacity during mission execution. This evidence-based approach to physical conditioning requires the systematic implementation of daily resistance training and compound exercise protocols [10,11,12].

Furthermore, a soldier’s physical fitness is reflected in several key areas, including good bodily health, the ability to execute both agile and prolonged movements, the capacity to recover quickly after intense exertion, the ability to carry out assigned tasks and missions, and the capacity to maintain confidence under all circumstances [10]. It is paramount to consider the various factors that influence the fitness level of military forces, given the necessity for optimal performance in diverse climatic conditions. Among the physiological determinants of performance, circadian rhythm patterns and individual chronotype classifications demonstrate particular significance. Consequently, elucidating the influence of circadian variations on strength parameters in military cadets carries direct implications for optimizing training periodization and enhancing operational readiness protocols [11]. Meta-analytical evidence indicates that operational physical activities induce transient decrements in specific physiological capacities, notably lower-limb power output and muscular endurance performance. These empirical findings emphasize the critical need for developing evidence-based training interventions to attenuate performance impairments and maintain optimal operational capacity [12]. Finally, contemporary research investigating the efficacy of structured physical training programs in augmenting tactical and combat preparedness among military personnel demonstrates that physical fitness constitutes a fundamental determinant for sustaining physiological vigour, cardiovascular endurance, and task-specific performance in military operational contexts [13].

From our knowledge, there are no studies linking circadian rhythms to variations in force production within military populations. Through a series of physical tests, this study aims to determine whether strength and power varies across the working day of military cadets.

## 2. Materials and Methods

**Subjects**: We recruited 43 cadets (n = 23 males and 20 females) from a local military base. There were no sex differences in age, while body mass index was higher for males (Table 1). A priori power analysis was performed using GPower. Based on the effect size for jump power between 09:00 a.m. and 02:30 p.m. reported in a previous study (Cohen’s d = 0.39; [14]), we estimated that 20 participants per group (men vs. women) would be required to achieve 90% power to detect a similar effect at an alpha level of 0.05.

They had to meet the following inclusion criteria: age between 20 and 50 years, at least two years of military experience involving daily military physical activities, ability to understand the study’s instructions, objectives, and protocol, and an intermediate chronotype (42–58 score on the Horne and Östberg questionnaire—reference). Chronotype is a biological trait that determines an individual’s preferred timing for daily activities, governed by the circadian system. It represents a continuum from early chronotype, where individuals exhibit an advanced sleep–wake cycle and peak activity in the morning, to late chronotype, characterized by delayed sleep onset and peak alertness in the evening. The exclusion criteria included a history of significant adverse cardiovascular events or smoking.

Volunteers who wished to participate in the study and met the inclusion criteria were provided with a specifically designed information letter. Any doubts were clarified before signing the informed consent form. All procedures were approved by the Ethical Committee of Universidad Loyola, Andalucía (9 September 2024).

**Study design**: All procedures were conducted at the military base. Initially, an explanatory session was given to inform about the study’s objectives and methodology, addressing any existing doubts, and administering the Horne and Ostberg test to determine each participant’s chronotype.

The subjects completed the Horne and Ostberg test [15], which assigns a quantitative value between 16 and 86 to chronotype quality. The following categories were established: 16–30 (Extreme Evening), 31–41 (Moderate Evening), 42–58 (Intermediate), 59–69 (Moderate Morning), and 70–86 (Extreme Morning). Only subjects with an Intermediate Chronotype were included in the present study.

Volunteers who met the inclusion and exclusion criteria were scheduled for three sessions within seven days, with at least 48 h between separate experimental days. During this period, they were instructed to avoid strenuous physical activity, including work-related activities. They were also asked to maintain their habitual nutritional habits, except for caffeine (coffee), which they were instructed to avoid on experimental days, and it was verified through self-report questionnaires before each session.

*First day*: Subjects were instructed about the protocols to perform during the experimental days. They were familiarized with all the tests, and anthropometric data were collected. This visit took place at the end of their working day.

*Experimental days*: Subjects were randomly assigned to the testing order using a computer-generated randomization list, to attend either at the beginning (08:30 a.m.) or at the end (02:30 p.m.) of their working day during the second (experimental) day. On the third day, the timing was reversed. After a ~15 min RAMP-type warm-up, they performed the physical tests. The mean of three attempts was used for analysis, with each attempt separated by 30 s to 1 min.

**Warm-up**: Subjects performed a RAMP-type warm-up, which consists of three phases: the Raise phase, the Activation/Mobilization phase, and the Potentiation phase [16].

*Raise Phase*: Seven exercises were performed over a straight 15 m distance, incorporating linear and multidirectional movements combined with dynamic upper limb mobility on the way out and jogging on the way back.

*Activation/Mobilization Phase*: Five dynamic mobility exercises targeting the ankle, knee, hip, and spine were performed for eight repetitions each (Prayer Squat, Lateral Lunge, Frontal Lunge and Turn, Single-leg Romanian deadlift and Quadriceps Stretch). Additionally, six activation exercises using mini-bands and small balls were performed for eight repetitions each (Wall Push-up, Squat with mini-band, Lateral Walk with mini-band, Monster Walk with mini-band, and hand grip with a ball first from the anatomical position and then with the arms extended sideways).

*Potentiation Phase*: Five Drop Jumps were performed from a 30 cm step, followed by pogo jumps over 15 m on both legs and one leg. The phase concluded with three Squat Jumps and three accelerations over 15 m.

**Squat Jump and Countermovement Jump Tests**: The Squat Jump (SJ) consists of a maximal jump from a flexed position with the hands on the hips. At the same time, the Countermovement Jump (CMJ) involves leg flexion from a standing position, immediately followed by a maximal jump with the hands off the hips. Jump height (cm) was recorded using the Optojump device [17]. In addition, we evaluated the difference (%) between CMJ and SJ (i.e., jump index) to evaluate the ability to utilize the stretch-shortening cycle during the CMJ.

**Handgrip Strength**: After adjusting the grip to fit the subject’s hand size, maximal muscle strength was measured using a hand dynamometer (TKK 5401 Grip D, Takey, Tokyo, Japan). Subjects were verbally encouraged to “squeeze as hard as possible” and to exert maximum effort for at least three seconds (s). The test was performed in a standing position, with the elbow fully extended and the wrist in a neutral position [18].

**Isometric Leg Extension**: We used the ActiveForce 2 (Activbody, San Diego, CA, USA) digital dynamometer to assess maximal isometric strength [19]. Subjects performed the test while seated on a massage table, with the dynamometer anchored with straps to the leg of the stretcher, positioning the knee at a 60° flexion angle, as previously described [20]. Subjects were instructed to apply pressure progressively for two seconds, after which they were encouraged to maintain maximum extension force for an additional three seconds. The researcher ensured that the dynamometer remained in the correct position at all times, holding it with both hands [20].

**Statistical Analysis**: All statistical analyses were performed using R (v. 4.2.2). Results are presented as mean ± SD. Comparisons in Table 1 (characteristics) were conducted using independent *t*-tests. The effects of sex and time of day were analyzed using a 2 (sex) × 2 (time) repeated-measures ANOVA. Across the set of dependent variables, *p*-values were adjusted using Holm–Bonferroni correction; effect sizes and confidence intervals are reported without adjustment. Data are expressed as mean ± SD, and a *p*-value of 0.05 was set as the criterion for statistical significance.

## 3. Results

The analysis of jump performance revealed clear sex differences but no significant effects of Time or Time × Sex interaction (all *p* > 0.26; Table 2). For the squat jump (SJ), men jumped on average 8.9 cm higher than women [95% CI: 5.8, 12.0], corresponding to a large effect size (Cohen’s d = 1.77). Although SJ performance tended to be slightly higher in the afternoon (Δ morning–afternoon = −1.19 cm [95% CI: −3.40, 1.03]), this effect was small and nonsignificant (*p* = 0.289). A similar pattern was observed for the countermovement jump (CMJ), where men exceeded women by 10.5 cm [95% CI: 8.6, 13.6; d = 1.86]. Time-of-day differences in CMJ were trivial (Δ = −1.30 cm [−3.74, 1.13], *p* = 0.289). The jump index was also higher in men (Δ = 1.6% [−0.9, 4.1], d = 0.59), with no evidence of variation across sessions (Δ = 0.09% [−1.91, 2.10], *p* = 0.927).

Strength outcomes confirmed these trends. Right and left leg extension strength were markedly greater in men (Δ = 132 N [95% CI: 101, 163], d = 1.78; and Δ = 128 N [92.9, 164], d = 1.59, respectively). However, neither variable showed significant time-of-day effects (ExtD: Δ = 6.83 N [−24.31, 37.96], *p* = 0.664; ExtI: Δ = −2.20 N [−37.57, 33.18], *p* = 0.902). For handgrip, men exhibited the largest sex differences, with 16.1 kg [95% CI: 13.8, 18.4] higher strength in the dominant hand (DH: d = 3.08) and 15.6 kg [13.2, 18.0] in the non-dominant hand (NDH: d = 2.94). Again, time-of-day differences were negligible (DH: Δ = −0.95 kg [−3.24, 1.34], *p* = 0.410; NDH: Δ = −0.86 kg [−3.24, 1.51], *p* = 0.471).

Overall, sex explained a substantial proportion of variance across all outcomes (partial η^2^ = 0.39–0.71), while time-of-day effects were consistently small (partial η^2^ < 0.02) and non-significant. These findings indicate robust sex-related differences in both jumping and strength performance, but no evidence that performance is influenced by the time of testing within the military working day. No adverse events were reported during testing.

## 4. Discussion

Many physiological variables follow a circadian rhythm, including skeletal muscle strength, which is known to peak in the afternoon (~06:00 p.m.) [21,22]. However, the implications of this strength-related circadian rhythmicity remain relatively unknown in populations engaged in daily physical activity throughout their working day.

In this study, we analyzed for the first time whether strength capacities vary during the working day in military cadets. Our main finding indicates that there is no evidence of significant variations in strength-related capacities when assessed early (08:30 a.m.) or late (02:30 p.m.) during the working day in military cadets. Additionally, we found that this effect is unlikely to differ between male and female cadets.

Chtourou et al. analyzed 30 male physical activity students at 07:00 a.m. and 05:00 p.m. Their results showed significantly higher SJ and CMJ performance in the afternoon. They also demonstrated that regular strength training at a specific time (morning or afternoon) enhances anaerobic performance at that particular time [23]. This may partially explain the discrepancies between their study and ours, as military cadets are required to engage in physical activity throughout their working hours. It is also possible that the narrow testing window for tests in the current study contributed to the lack of differences between early and late jumping capacity. However, previous research has shown that, compared to 09:00 a.m., jump performance is higher at 02:00 p.m., with no differences between 02:00 p.m. and 06:00 p.m. [14].

The divergence of our findings from previous reports in athletic and general cohorts—where diurnal variations in strength or power have been observed—can be attributed to methodological discrepancies, notably in pre-test warm-up procedures. Whereas earlier investigations [14,23] often employed non-standardized or insufficiently activating warm-up routines, our protocol was specifically designed to optimize neuromuscular readiness and thereby mitigate circadian-related performance fluctuations. Consequently, the lack of time-of-day effects in the present study may likely reflect the efficacy of a rigorously standardized warm-up strategy in stabilizing neuromuscular performance across diurnal phases. In this regards, longer warm-ups have been suggested to minimize diurnal fluctuations in anaerobic performance [24]. Studies investigating jump and anaerobic performance across the day often fail to precisely define warm-up protocols or use non-specific warm-ups (e.g., cycling) [14,23]. Since cadets have limited time during their working day, we chose not to extend the duration of the warm-ups. Instead, we implemented a RAMP warm-up, which is known to effectively increase muscle and core temperature, as well as neuromuscular activity [16] in a short time. Therefore, the discrepancies between our data and studies showing diurnal variations in strength and power performance when testing at 08:00 a.m. and 02:00 p.m. [14] may be attributed to differences in warm-up protocols.

The absence of circadian variation in strength performance among cadets may also be attributed to their habitual engagement in daily physical activity and adherence to regimented routines, as well as the implementation of the standardized RAMP warm-up protocol as discussed earlier. A further methodological strength of the present study is the inclusion of chronotype assessment for each participating military subject, thereby enabling a comprehensive analysis of individual circadian predispositions concerning performance outcomes [23]. This investigation is subject to limitations. The relatively small sample size (n = 43) constrains the generalizability of the results, and expanded cohorts encompassing greater diversity in military populations, age, and sex are necessary to validate the absence of circadian variation. The temporal scope of testing was confined to sessions at 08:30 and 14:30, precluding assessment during the evening peak (~18:00) frequently documented in the literature; as such, the complete circadian profile was not addressed. Furthermore, the lack of hormonal and physiological measurements (e.g., cortisol, testosterone, core temperature) restricts the mechanistic interpretation of the observed outcomes. Lastly, this study focused exclusively on strength and jump performance outcomes, whereas domains such as endurance, cognitive function, and injury risk warrant exploration in subsequent research.

## 5. Conclusions

When a standardized warm-up specifically designed to optimize neuromuscular activation is applied, strength and power performance in military cadets remain stable across the working day, with no meaningful differences observed between morning and afternoon testing sessions. This stability was evident across all measures, including vertical jumps, leg extension strength, and handgrip strength, and was consistent for both male and female cadets with an intermediate chronotype. These results suggest that, under controlled warm-up conditions, circadian variations in neuromuscular performance may be minimized, even in highly physically active populations such as military personnel.

## Figures and Tables

**Table 1:** Characteristics of the subjects.

	Females (n = 20)	Males (n = 23)	*p*-Value
**Age**	30.8 ± 7.1	29.8 ± 8.7	0.533
**BMI**	23.5 ± 2.6	25.5 ± 2.3	0.01 *
**Chronotype #**	53.3 ± 3.7	52.4 ± 4.4	0.438

* Significantly lower than 0.05. BMI: Body mass Index. # Score from the Horne and Ostberg questionnaire.

**Table 2:** Jumping and strength analysis for sex, time and time × sex interaction.

	Females Early (n = 20)	Females Late (n = 20)	Males Early (n = 23)	Males Late (n = 23)	Sex (*p*)	Time (*p*)	Sex × Time (*p*)	Δ H–M [95% CI]	Cohen’s d [95% CI]	Partial η^2^ [95% CI]
**SJ (cm)**	21.3 ± 3.8	21.7 ± 3.8	29.5 ± 6.0	31.4 ± 6.2	<0.001	0.268	0.522	8.9 [5.8, 12.0]	1.77 [1.38, 2.34]	0.44 [0.31, 1.00]
**CMJ (cm)**	22.7 ± 4.2	23.2 ± 4.6	32.3 ± 6.3	34.3 ± 6.9	<0.001	0.27	0.552	10.5 [8.6, 13.6]	1.86 [1.51, 2.39]	0.47 [0.34, 1.00]
**JI (%)**	6.0 ± 5.1	6.0 ± 5.5	8.8 ± 4.7	8.6 ± 3.2	0.008	0.919	0.893	1.6 [−0.9, 4.1]	0.59 [0.17, 0.99]	0.02 [0.00, 1.00]
**RL strength (N)**	342 ± 56.8	342 ± 69.1	475 ± 76.2	462 ± 82.5	<0.001	0.664	0.664	132 [101, 163]	1.78 [1.36, 2.29]	0.47 [0.34, 1.00]
**LL strength (N)**	340 ± 65.5	338 ± 71.2	464 ± 84.4	470 ± 100	<0.001	0.902	0.824	128 [92.9, 164]	1.59 [1.18, 2.08]	0.39 [0.26, 1.00]
**DH strength (kg)**	33.4 ± 4.2	34.0 ± 4.8	49.1 ± 6.2	50.5 ± 5.7	<0.001	0.41	0.73	16.1 [13.8, 18.4]	3.08 [2.46, 3.96]	0.71 [0.62, 1.00]
**NDH strength (kg)**	33.2 ± 4.3	34.1 ± 4.4	48.9 ± 5.8	49.6 ± 6.6	<0.001	0.471	0.92	15.6 [13.2, 18.0]	2.94 [2.43, 3.72]	0.68 [0.59, 1.00]

Values are mean ± SD. Δ H–M = mean difference between men and women, with 95% confidence intervals. Cohen’s d (95% CI) refers to the standardized mean difference between sexes (main effect). Partial η^2^ (95% CI) corresponds to the effect size of the Sex factor from the two-way ANOVA. *p*-values for the main effects of Sex, Time, and their interaction are shown, adjusted for multiple comparisons using Holm–Bonferroni correction across variables. CMJ, countermovement jump; DH, dominant hand; JI, jump index; NDH, non dominant hand; LL, isometric left leg extension; SJ, squat jump; RL, isometric right leg extension.

## Data Availability

The original contributions presented in this study are included in the article. Further inquiries can be directed to the corresponding author (rcasuso@uloyola.es).
